# Machine Learning-Based Early Prediction of Sepsis Using Electronic Health Records: A Systematic Review

**DOI:** 10.3390/jcm12175658

**Published:** 2023-08-30

**Authors:** Khandaker Reajul Islam, Johayra Prithula, Jaya Kumar, Toh Leong Tan, Mamun Bin Ibne Reaz, Md. Shaheenur Islam Sumon, Muhammad E. H. Chowdhury

**Affiliations:** 1 Department of Physiology, Faculty of Medicine, University Kebangsaan Malaysia, Kuala Lumpur 56000, Malaysia; 2 Department of Electrical and Electronics Engineering, University of Dhaka, Dhaka 1000, Bangladesh; 3 Department of Emergency Medicine, Faculty of Medicine, Universiti Kebangsaan Malaysia, Kuala Lumpur 56000, Malaysia; 4 Department of Electrical and Electronic Engineering, Independent University, Bangladesh Bashundhara, Dhaka 1229, Bangladesh; 5 Department of Biomedical Engineering, Military Institute of Science and Technology (MIST), Dhaka 1216, Bangladesh; 6 Department of Electrical Engineering, Qatar University, Doha 2713, Qatar

**Keywords:** sepsis, machine learning, deep learning, early prediction, electronic health record, intensive care unit (ICU), emergency department (ED)

## Abstract

Background: Sepsis, a life-threatening infection-induced inflammatory condition, has significant global health impacts. Timely detection is crucial for improving patient outcomes as sepsis can rapidly progress to severe forms. The application of machine learning (ML) and deep learning (DL) to predict sepsis using electronic health records (EHRs) has gained considerable attention for timely intervention. Methods: PubMed, IEEE Xplore, Google Scholar, and Scopus were searched for relevant studies. All studies that used ML/DL to detect or early-predict the onset of sepsis in the adult population using EHRs were considered. Data were extracted and analyzed from all studies that met the criteria and were also evaluated for their quality. Results: This systematic review examined 1942 articles, selecting 42 studies while adhering to strict criteria. The chosen studies were predominantly retrospective (n = 38) and spanned diverse geographic settings, with a focus on the United States. Different datasets, sepsis definitions, and prevalence rates were employed, necessitating data augmentation. Heterogeneous parameter utilization, diverse model distribution, and varying quality assessments were observed. Longitudinal data enabled early sepsis prediction, and quality criteria fulfillment varied, with inconsistent funding–article quality correlation. Conclusions: This systematic review underscores the significance of ML/DL methods for sepsis detection and early prediction through EHR data.

## 1. Introduction

Sepsis is a potentially fatal illness that occurs when the body’s response to an infection causes tissue and organ damage [1]. It is a complex syndrome characterized by a dysregulated host immune response, organ dysfunction, and a high risk of mortality [2]. Sepsis can affect people of all ages, from infants to the elderly, and can arise from various types of infections, including bacterial, viral, and fungal infections [3]. When an infection occurs, the body’s immune system releases chemicals to combat the invading pathogens. In sepsis, the immune response becomes dysregulated, leading to widespread inflammation throughout the body. This inflammatory response can damage tissues and impair organ function [4]. Sepsis can develop into severe sepsis or septic shock, which are life-threatening illnesses with high fatality rates if ignored [5]. Fever, a faster heartbeat, rapid breathing, changed mental status, decreased urine output, low blood pressure, and general malaise are some of the symptoms of sepsis that might vary [6]. An intensive care unit (ICU) must provide urgent medical care and treatment for sepsis [7]. The standard treatment involves administering both intravenous antibiotics to combat the underlying infection as well as intravenous fluids to maintain adequate blood pressure and organ perfusion [8]. In severe cases, additional interventions such as vasopressor medications to raise blood pressure, mechanical ventilation to support breathing, and renal replacement therapy may be necessary [9].

Sepsis imposes a substantial burden on healthcare systems worldwide. Globally, it stands as a leading cause of illness and mortality, incurring substantial treatment expenses and leaving survivors with lasting repercussions [10,11,12]. Sepsis significantly elevates mortality rates. Fleischmann et al. (2016) conducted a systematic review and meta-analysis and found that there are about 48.9 million cases of sepsis every year, with a 19.4% death rate [13]. The recorded death rate was about 15.7% in high-income countries, but it was much higher in low- and middle-income countries, where it stood at 34.7%. Angus et al. found in 2001 that the death rate for serious sepsis and septic shock is between 40 and 60% [10]. Long-term physical, cognitive, and psychological impairments are common among survivors of sepsis, resulting in a significant burden of morbidity. The Surviving Sepsis Campaign (SSC) conducted a study involving over 1000 sepsis survivors and discovered that one year after discharge, 33% of patients had cognitive dysfunction, 43% had new functional limitations, and 27% had symptoms of post-traumatic stress disorder (PTSD) [14]. These long-term complications can have significant impacts on survivors’ quality of life and result in substantial healthcare requirements. Sepsis places a significant burden on healthcare resources, increasing hospitalizations, ICU stays, and healthcare costs. According to a study by Rudd et al. (2013) [10], the total annual cost of sepsis hospitalizations in the United States was approximately $24 billion, or 13.3% of all hospital expenditures. Patients with sepsis require intensive monitoring, invasive procedures, and broad-spectrum antibiotics, all of which increase the utilization of healthcare resources. In addition, sepsis has been linked to an increased risk of readmissions and hospital-acquired infections, which further strains healthcare systems. Rhee et al. (2017) [15] found in a retrospective cohort study that sepsis survivors had a 38% higher risk of hospital readmission within 90 days compared to non-sepsis patients. Not only do these readmissions increase healthcare costs, but they also add to the overall burden on hospitals and healthcare facilities. For healthcare providers, sepsis presents significant challenges, including the need for prompt diagnosis, intervention, and the management of complications. The complexity and unpredictability of sepsis necessitate a multidisciplinary approach and place a significant burden on healthcare teams. The emotional toll of caring for critically ill septic patients, high mortality rates, and the risk of healthcare provider burnout are emerging concerns [12]. Providing healthcare providers with the necessary resources, support systems, and education is essential to addressing these challenges and improving patient outcomes. 

Early identification and rapid treatment are, therefore, essential for enhancing patient outcomes. Prevention of sepsis involves measures such as proper hygiene practices, vaccination against infectious diseases, the prompt treatment of infections, and the appropriate use of antibiotics [16]. Additionally, the early identification of individuals at risk, such as those with compromised immune systems or chronic medical conditions, can help in implementing preventive strategies and prompt treatment [17].

In recent decades, data-driven biomarker discovery has garnered traction as an alternative to conventional methods with the potential to overcome existing obstacles. This strategy seeks to harvest and exploit health data using quantitative computer methods like machine learning. High-resolution digital data are becoming more available to persons at risk and patients with sepsis [18]. These include laboratory, vital, genetic, genomic, clinical, and health history data. Improved patient outcomes can be achieved with the early detection and prediction of sepsis [19]. In recent years, machine learning (ML) and deep learning (DL) algorithms have shown promise in sepsis diagnosis and early prediction [20] by analyzing large-scale patient data. These algorithms can be used with electronic health records (EHRs) and other clinical data to identify patterns and indicators of sepsis that may not be immediately apparent to human practitioners [21]. This enables the early detection of sepsis and the beginning of prompt therapies. 

Machine learning techniques have greatly aided in sepsis detection and prediction. Logistic regression, support vector machines (SVMs), random forests, and gradient boosting are examples of common machine learning approaches. These algorithms make use of vital signs (such as heart rate and blood pressure), laboratory results (such as white blood cell count and lactate level), and clinical factors (such as age and comorbidities) to predict the likelihood of sepsis. High sensitivity, specificity, and area under the receiver operating characteristic curve (AUC-ROC) values have been reported for these models by researchers, demonstrating their propensity for sepsis prediction [18,22,23,24,25,26,27,28,29]. In response to the persisting challenge of sepsis-related fatalities, Wang et al. [30] sought to develop an AI algorithm for early sepsis prediction, successfully creating a random forest model utilizing 55 clinical features from ICU patient data, yielding an AUC of 0.91, 87% sensitivity, and 89% specificity, with potential wider applicability pending external validation. Similarly, Kijpaisalratana et al. (2022) [31] have developed novel machine learning-based sepsis screening tools and compared their performance with traditional methods for early risk prediction of sepsis. Using retrospective electronic health record data from emergency department visits, the machine learning models, including logistic regression, gradient boosting, random forest, and neural network models, exhibited significantly better predictive performance (e.g., AUROC 0.931) compared to reference models (AUROC 0.635 for qSOFA (quick Sepsis Related Organ Failure Assessment), 0.688 for MEWS (Modified Early Warning Score), and 0.814 for SIRS (Systemic Inflammatory Response Syndrome)), highlighting their potential to enhance sepsis diagnosis in emergency patients. The ability of DL, a subset of ML, to automatically develop hierarchical representations from complicated data has generated a great deal of interest in sepsis prediction. DL models, particularly convolutional neural networks (CNNs) and recurrent neural networks (RNNs) have shown encouraging results in several medical applications, including in sepsis detection. Nemati et al. (2018) combined CNN and long short-term memory (LSTM) networks to construct a deep learning model to predict sepsis using EHR data. The model beat traditional ML models with an AUC-ROC of 0.83 [32]. Addressing the pressing issue of sepsis’s exponential impact and increased mortality in ICU patients, Singh et al. [33] presents a highly accurate machine learning model for early detection, outperforming existing approaches with a proposed ensemble model achieving a balanced accuracy of 0.96.

This article’s goal is to give readers an overview of recent studies on ML- and DL-based sepsis diagnosis and early prediction. Due to the rapid pace of progress in this field of study, it is essential to review and evaluate the present state of the art in the field of sepsis detection and onset prediction. By analyzing the pertinent research, we assessed the effectiveness of various ML and DL models, the features employed for prediction, and the difficulties and future directions in this area. The purpose of this research was to provide an in-depth investigation of the most recent research by utilizing data taken from the electronic health records of adults and employing ML and DL models. To achieve this, we thoroughly searched for relevant articles in four popular electronic databases and developed a new assessment criterion for the quality assessment of different articles. The characteristics of different selected articles are thoroughly studied and summarized in this article. To the best of our knowledge, this article covers a systematic review of the largest number of research articles published between Jun 2016 and March 2023. In addition, we have discussed the current challenges and future direction of research in this domain, along with the limitations of the current study.

## 2. Background and Fundamental Concepts

The subsequent portion of this section provides concise explanations of key concepts that are essential for a deeper comprehension of the subjects addressed in the following sections. It is important to note that the selection of methods discussed in this section mirrors the methodologies utilized in the chosen articles as a direct outcome of the systematic review process.

### 2.1. Sepsis Definition

Sepsis criteria and definitions have changed over time to improve early care and identification. Sepsis-2 and Sepsis-3 are the two main definitions of sepsis that have gained widespread support.

The Sepsis-2 definition [34] is based on the existence of a systemic inflammatory response syndrome (SIRS) brought on by infection and was first published in 2001. According to Sepsis-2, sepsis is characterized by the presence of at least two SIRS criteria, which include leukopenia or leukocytosis in the case of abnormal white blood cell counts, as well as abnormal body temperature (fever or hypothermia), increased heart rate (tachycardia), increased respiratory rate (tachypnea), and abnormal body temperature (fever or hypothermia). Septic shock is defined as severe sepsis with prolonged hypotension despite fluid resuscitation, and severe sepsis combined with organ dysfunction is known as severe sepsis.

The Sepsis-3 definition [35] was put up in 2016 to increase the precision and clinical utility of sepsis detection. Instead of depending primarily on the SIRS criteria, it emphasizes organ failure as sepsis’ defining feature. According to Sepsis-3, sepsis is characterized as a potentially fatal organ malfunction brought on by an improperly managed host defense against infection. The Sequential Organ Failure Assessment (SOFA) score, which assesses the function of the respiratory, cardiovascular, hepatic, renal, coagulation, and central nervous systems, is used to measure organ dysfunction. If the SOFA score rises by two or more points as a result of infection, sepsis is thought to be present. A blood lactate level of more than two mmol/L and persistent hypotension requiring vasopressors to maintain a mean arterial pressure of 65 mmHg or higher are both considered signs of septic shock. These definitions play a crucial role in standardizing the identification and management of sepsis, aiding clinicians in making timely and accurate diagnoses and facilitating effective interventions to improve patient outcomes. The most widely adopted definition of sepsis is Sepsis-3 [36]. The key advantages of the Sepsis-3 definition include the following:

- By incorporating organ dysfunction criteria, the Sepsis-3 definition improves the specificity of sepsis diagnosis. This helps differentiate sepsis from other conditions that may present with signs of infection but do not involve organ dysfunction.
- The Sepsis-3 definition simplifies the criteria for sepsis by focusing on organ dysfunction rather than the systemic inflammatory response syndrome (SIRS) criteria used in previous definitions. This simplification reduces the potential for misdiagnosis and facilitates a more targeted approach to sepsis identification.
- The early prediction of sepsis is crucial for timely intervention. The SOFA score, which is part of the Sepsis-3 definition, provides a tool for assessing organ dysfunction and predicting patient outcomes. Higher SOFA scores are associated with increased mortality rates and can help identify patients at higher risk who require immediate attention.
- The Sepsis-3 definition has facilitated the standardization of sepsis diagnosis and research. The use of a consistent definition enables better comparison of studies, data sharing, and the development of evidence-based management strategies.

### 2.2. Common Machine Learning Models and Performance Metrics

A summary of common classical machine learning (ML) and deep learning models, as well as of performance metrics commonly used for the early prediction of sepsis, is listed below.

Classical ML Models:
  - Decision Trees (DT)
  - Random Forest (RF)
  - Support Vector Machine (SVM)
  - Logistic Regression (LR)
  - Gradient Boosting (GB)
  - Naïve Bayes (NB)
  - k-Nearest Neighbor (kNN)

Deep Learning Models:
  - Long Short-Term Memory (LSTM) Networks
  - Convolutional Neural Network (CNN)
  - Gated Recurrent Unit (GRU)
  - Neural Network (NN)
  - Multitask Gaussian Process and Attention-based Deep Learning Model (MGP-AttTCN)
  - Temporal Convolutional Network (TCN)
  - Recurrent Neural Network (RNN)
  - CNN-LSTM
  - CNN-GRU

Performance Metrics:
  - Area Under the Curve (AUC) or AUROC (Receiver Operating Characteristics Curve)
  - Sensitivity (Recall)
  - Specificity
  - Accuracy
  - Precision
  - F1 score
  - Matthews Correlation Coefficient (MCC)
  - Mean Average Precision (mAP)
  - Positive Predictive Value (PPV)
  - Negative Predictive Value (NPV)
  - Positive Likelihood Ratio (PLR)
  - Negative Likelihood Ratio (NLR)

These models and metrics have been widely used to assess the predictive performance of algorithms for early sepsis prediction using EHRs. The selection of models and metrics may vary depending on the specific study and dataset under consideration. Supplementary Tables S1 and S2 provide short definitions of these ML and DL models and performance metrics.

## 3. Materials and Methods

### 3.1. Search Strategy 

**Figure 1:**
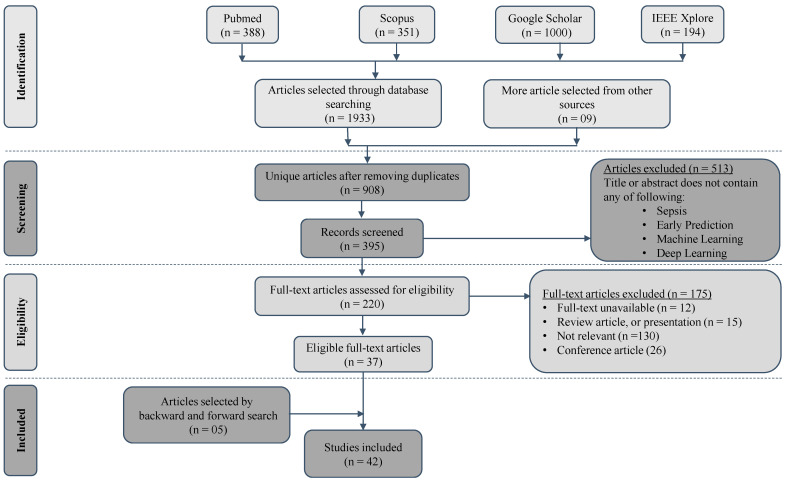
Search and selection process using PRISMA flowchart.

A comprehensive search strategy was employed to identify relevant studies from electronic databases including PubMed, IEEE Xplore, Google Scholar, and Scopus (Figure 1). The bibliographic research for this systematic review was conducted by a team of two experienced researchers with expertise in the fields of medical informatics and machine learning. Additionally, the team consulted an information specialist with extensive knowledge in database searching and retrieval. The search terms used included variations of “sepsis”, “prediction”, “machine learning”, “deep learning”, and “electronic health records”. The search was limited to articles published in English between Jun 2016 and Mar 2023. This systematic review is not registered in PROSPERO or any other database. 

We combined our search terms using Boolean operators (e.g., AND, OR) to create an effective search query. We used parentheses to group related terms: for example, “(sepsis OR prediction) AND (machine learning OR prediction) AND (electronic health records OR prediction)”. We executed our search query in each selected database. We applied the necessary filters, such as publication date or language, to refine our results, and saved the search strategy for reporting purposes, as shown in Supplementary Table S3. 

### 3.2. Inclusion and Exclusion Criteria

The following inclusion and exclusion criteria were used for this study: 

#### 3.2.1. Inclusion Criteria

- Studies that focused on the application of machine learning and deep learning algorithms for the early prediction of sepsis.
- Studies utilizing electronic health records (EHR) data as the primary sources of information.
- Studies that involved adult human subjects (i.e., age ≥ 18).
- Studies that reported on the performance metrics (e.g., sensitivity, specificity, area under the curve) of the machine learning models for sepsis prediction.
- Studies published in peer-reviewed journals.
- Studies available in the English language.
- Studies published within a specific time frame (e.g., Jun 2016 and March 2023).

#### 3.2.2. Exclusion Criteria

- Studies that did not focus on early prediction of sepsis.
- Studies that focused only on using clinical notes.
- Studies that did not involve the use of machine learning/deep learning algorithms.
- Studies that did not utilize electronic health records as data sources.
- Studies that primarily focused on non-human subjects or experimental setups not related to human healthcare.
- Studies that did not report on performance metrics for machine learning models.
- Studies that were not published in peer-reviewed journals.
- Studies published in languages other than English.

It should be noted that Sepsis definition was not considered in the inclusion or exclusion criteria. The Population, Intervention, Comparator, Outcome, and Study Design (PICOS) criteria for the systematic review, including both the inclusion and exclusion criteria, are detailed in Supplementary Table S4.

### 3.3. Study Selection

After retrieving search results using the above-mentioned search strategy in the selected databases, we used manual screening to identify and remove duplicate articles from the search results. We reviewed the titles and abstracts of the remaining articles to determine their relevance to our research question and inclusion/exclusion criteria. We excluded clearly irrelevant articles at this stage. We excluded randomized controlled trials, study overviews and protocols, and meta-epidemiological studies. We also checked the systematic reviews and meta-analysis papers published in the time span and cross-checked them with what we had collected, and added any missing article as “More article selected from other sources”.

The study selection process was carried out by two independent authors, and discrepancies were resolved through discussion and consensus. In cases where disagreements arose, a third author was consulted to make a final decision. The authors assessed the relevance of each study based on predetermined inclusion and exclusion criteria. The initial screening involved reviewing the titles and abstracts of the identified articles, followed by a full-text assessment of potentially eligible studies. This process was conducted in duplicate to ensure a thorough and unbiased selection of studies for inclusion in the systematic review. The inter-rater agreement between the two authors was assessed using Cohen’s Kappa coefficient, and a score of above 0.85 indicated substantial agreement. We aimed to minimize bias and ensure the robustness of study selection through this rigorous and collaborative process. 

### 3.4. Data Extraction

Data extraction was carried out independently by two authors, and any disparities were resolved through consensus. In cases where differences persisted, a third author was consulted to reach a final decision. The authors utilized a standardized data extraction form to systematically collect relevant information from the included studies. This process involved extracting key details such as study characteristics, participant demographics, intervention specifics, outcome measures, and relevant results. The data extraction was performed in duplicate to ensure accuracy and reliability. The level of agreement between the two authors was assessed using Cohen’s Kappa coefficient, with a value exceeding 0.85 indicating substantial concordance. We prioritized consistency and quality in data extraction through this collaborative and meticulous approach. To assess the quality of the articles and perform our systematic review, we extracted the following data from each article:

*Publication characteristics:* Collected the last name of the first author and the year of publication. 

*Study Design:* Identified the study design used in the article (e.g., prospective cohort study, retrospective analysis).

*Objectives:* Determined the specific research objectives or aims stated by the authors, which were supposed to match our study criteria.

*Cohort Selection:* Extracted information about the characteristics of the study participants such as sample size, demographics, prevalence of sepsis, and any relevant inclusion/exclusion criteria.

*Data Source*: Identified the source of the data used in the study (e.g., electronic health records, administrative databases, clinical trials) and whether it is a publicly accessible dataset or not.

*Model Selection*: Extracted information about machine learning or deep learning methods employed for data analysis, including feature engineering for classical ML, hyperparameters, ways to handle overfitting, etc.

*Reproducibility:* Assessed whether the article provides sufficient information to reproduce the study, including details on data availability, code availability, and software/hardware specifications.

*Performance Measures and Explainability*: Determined whether the performance matrices are reported in the study or not and whether the reported model is explainable or not.

*Limitations:* Noted the limitations or potential biases acknowledged by the authors in the article, such as sample size limitations, selection bias, or confounding factors.

*Funding Source*: Identified any sources of funding or financial support disclosed by the authors. 

### 3.5. Quality Assessment

The risk of bias in the included studies was assessed using a predefined comprehensive approach to assessing the quality of eligible articles. The criteria include unmet needs, reproducibility, robustness, generalizability, and clinical significance. Based on Moor et al. [18] and Qiao et al. [37], modified quality assessment criteria are presented, which include sample size (>50), data availability, code availability, mobile/web deployment, handling of missing data, sepsis prevalence, feature engineering, machine learning model, hyperparameters, overfitting prevention technique, reporting of performance metrics, validation using external data, explainability, limitation of the study in question, and discussion of clinical application. The inclusion of 16 relevant criteria and the use of a quality assessment table with “yes” or “no” ratings for each category contribute to a systematic and transparent evaluation of study quality. 

The quality assessment of the included studies was conducted by two authors independently, and any discrepancies were resolved through discussion and consensus. If a consensus could not be reached, a third author was involved to make a final decision. To evaluate inter-rater agreement, Cohen’s Kappa coefficient was computed, yielding a substantial agreement level of 0.85 between the two authors. This demonstrated a high level of concordance in their assessments. We placed significant emphasis on evaluating the methodological quality of the included studies, aiming to ensure the reliability and validity of the overall findings.

### 3.6. Impact of Funding Source

The funding source should not influence the design, conduct, analysis, interpretation, or reporting of a systematic review. It is crucial to maintain the independence and objectivity of the review process to ensure the integrity of the findings. Depending on the funding source, there may be implications for the generalizability of the findings. If the funding is specific to a particular setting, population, or intervention, it is important to consider the applicability of the results to other contexts. 

## 4. Results 

### 4.1. Selection Process

The initial search yielded a total of 1942 articles (Figure 1). Out of these initially selected articles, only 42 articles met the complete inclusion criteria. Most studies (n = 1900) were disregarded because they did not meet the inclusion criteria. These criteria included: research not involving machine learning or deep learning; conducting research on the wrong population (e.g., pediatric, or neonatal); conducting research on a topic outside the scope of the current review (e.g., mortality prediction); and conducting research using a study design different from that used in the current article, i.e., not peer reviewed.

### 4.2. Study Characteristics

**Figure 2:**
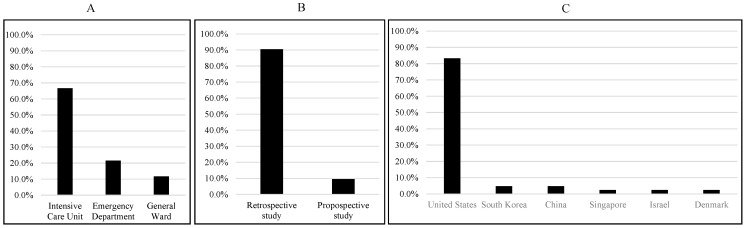
Summary of data sources (**A**), study types (**B**), and study populations (**C**).

Among the 42 studies selected for this systematic review, 38 were retrospective studies, while 4 were prospective studies. Most of the studies (35) use populations from the United States, while two studies are from China, two are from South Korea, and one each is from Singapore, Israel, and Denmark. The majority of the studies (34) used ICU data, while 11 studies used ED data, and the remaining six studies used general ward data (Figure 2). Some studies used data from multiple sources.

**Figure 3:**
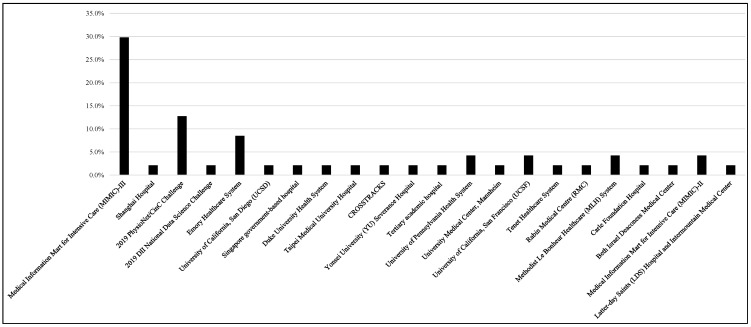
Distribution of data sources used to develop machine learning models.

Figure 3 shows that the most commonly used data source is MIMIC-III (n = 14, 29.8%), while the second most used dataset is the 2019 PhysioNet/CinC Challenge (n = 6, 12.8%), and the Emory Healthcare System (n = 4, 8.5%) is the third most used dataset. Each of the University of Pennsylvania Health System, University of California, San Francisco (UCSF), Methodist Le Bonheur Healthcare (MLH) System, and MIMIC-II datasets were used for two studies. It is worth noting here that some studies used multiple datasets. The Sepsis-2 (33.3%, n = 14) or Sepsis-3 (52.4%, n = 22) definitions of sepsis were utilized in the majority of the investigations. Depending on the nature of these studies, the time windows that were used have changed, and numerous researchers revised the Sepsis-2 or -3 definition. ICD-9 was utilized in certain studies (n = 4, 9.5%), while ICD-10 was used in others (n = 2, 4.8%). Intensive care unit specialists diagnosed sepsis in some of these investigations.

**Figure 4:**
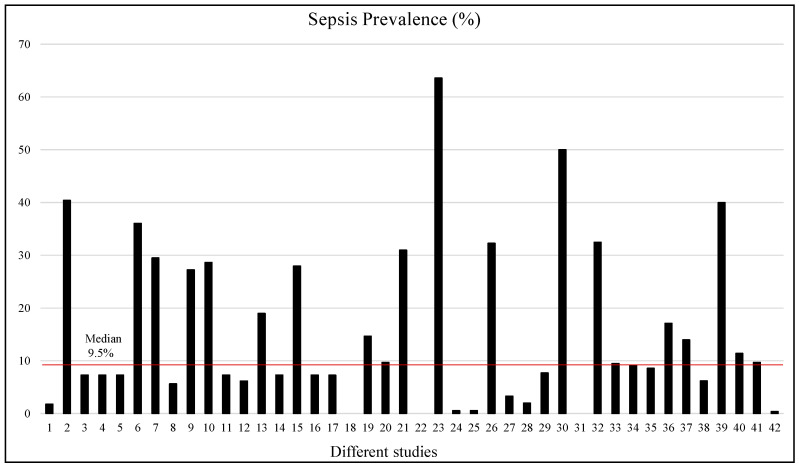
Sepsis prevalence (%) in different selected studies. The red line shows the median prevalence.

The proportion of patients with sepsis varied from 0.41% to 63.6%; however, most of the studies used imbalanced datasets, with very small numbers of patients with sepsis compared to the study populations. Figure 4 shows that in only 12 studies out of 42 studies, the prevalence of sepsis was more than 20%, while the median of sepsis prevalence was 9.5%. For three studies (18th, 22nd, and 31st), the sepsis prevalence data were not provided. Therefore, most of the ML or DL studies need to adopt some form of data augmentation or balancing techniques to develop reliable machine learning models. This systematic review only focused on the studies carried out on adult patients. Supplementary Figure S1 shows the sample size and sepsis-positive population in numbers and percentages, which made it possible to have a clear visual depiction of the imbalance distribution of the dataset. Two studies were discarded from this plot as those studies would make the plot biased, and a clear picture of the sample size and sepsis prevalence becomes unclear if those studies are incorporated.

**Figure 5:**
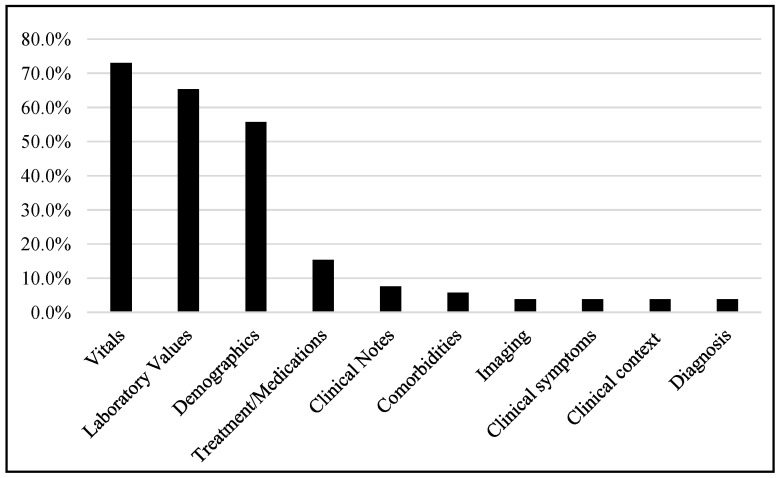
Different data types used in different studies.

Most of the studies were carried out using vital signs (73.1%), laboratory data (65.4%), and demographics (55.8%), while some studies also used clinical notes, treatments or medications, comorbidities, imaging, clinical context, diagnosis, etc., as shown in Figure 5. 

**Figure 6:**
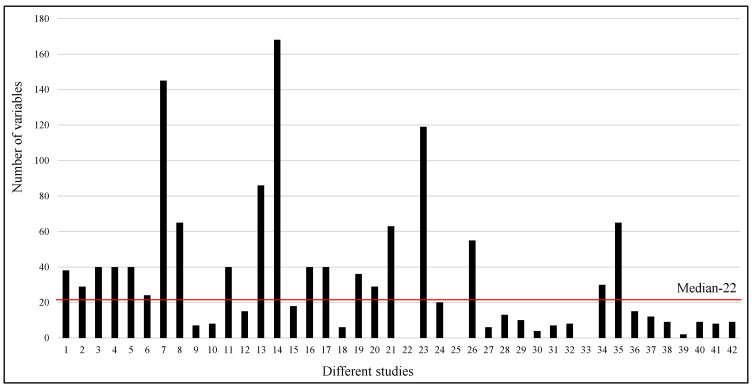
Number of variables used in different studies. The red line shows the median of the number of variables used in different studies.

A wide range of parameters is used in different studies, from as low as two to as high as one hundred and sixty eight. However, the median number of parameters used in the studies is 22. Figure 6 clearly shows that eight studies used more than fifty parameters, while most of the studies used a smaller number of parameters. Sixteen studies reported the feature importance among the variables available in the respective datasets.

**Figure 7:**
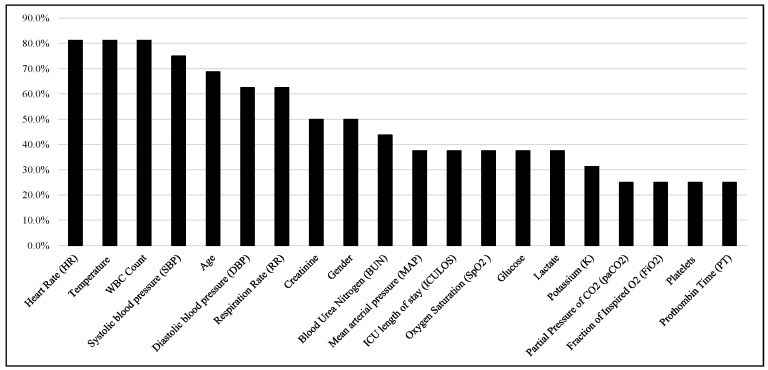
Top-ranked 20 features among the studies.

After the careful evaluation of important features identified by the studies, the Top 20 features were shown in Figure 7 based on their appearance in the important feature lists of 16 articles. It is noticed that heart rate, temperature, white blood cell (WBC) count, SBP, age, DBP, and RR are the six most ranked features reported in these studies.

### 4.3. Machine Learning Models

The selected studies exhibited a diverse array of machine learning and deep learning models, encompassing a total of 29 different approaches. Notably, within this spectrum, 58% of the articles opted for classical machine learning models, while the remaining 42% delved into the application of various deep learning models. This distribution underscores the comprehensive exploration of both traditional and cutting-edge techniques to address the complexities of early sepsis prediction.

### 4.4. Cross-Validation (CV) Techniques

Two cross-validation strategies were used in the articles: train-val-test split and N-fold cross-validation. Among the 42 selected articles, 14 articles used train-val-test split techniques, while 23 articles reported N-fold cross-validation techniques. Four different types of N-fold CV techniques are used: 4-fold CV (n = 4), 5-fold CV (n = 6), 6-fold CV (n = 1), and 10-fold CV (n = 12). It is clear that a 10-fold CV is popular among researchers for CV, while a large number of articles used a train-val-test split, which is equivalent to a single-fold CV.

### 4.5. Performance Metrics

Although different articles used different performance metrics, some performance metrics were common in the majority of articles, such as area under the curve (AUC) or area under the receiver operating characteristics curve (AUROC) (29.8%), sensitivity or recall (R) (19.8%), specificity (S) (21.5%), and accuracy (A) (9.9%). One metric was specific to the dataset, such as in the example of the Utility score being suitable for the 2019 PhysioNet/CinC Challenge. Other used metrics were precision, F1 score, F2 score, MCC, mAP, PPV, NPV, PLR, NLR, and response rate. AUROC values range between 0.80 and 0.97 in different studies. However, comparing model performance based solely on specific metrics can be limiting due to dataset variations. Given diverse datasets and experimental setups, assessing article performance solely based on such metrics is inappropriate. The models demonstrated improved accuracy and performance compared to traditional methods in identifying patients at risk of sepsis. Several studies reported high sensitivity and specificity, highlighting the potential for early detection and timely intervention. We have reported them in Table 1; however, we should not judge these articles based on these metrics. Moreover, it is beyond the scope of this systematic review to evaluate the articles using these performance metrics; rather, we have reported a quality evaluation in Section 3.5 to evaluate the quality of the articles based on 16 performance measures.

### 4.6. Early Prediction of Sepsis Onset

**Figure 8:**
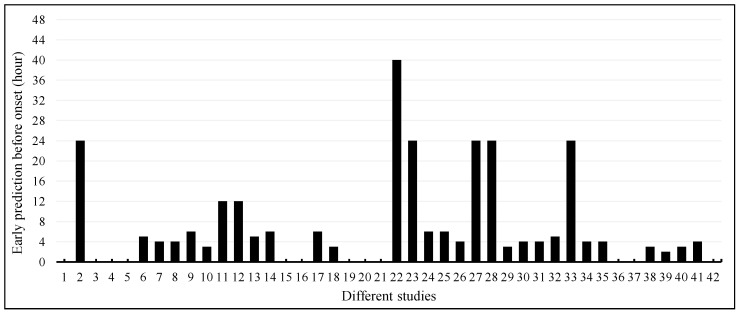
Early prediction of sepsis onset among different studies.

Among the 42 articles, 30 (71.4%) articles used longitudinal data for the early prediction of sepsis onset, while the remaining 12 (28.6%) articles did not report early prediction (Figure 8). Only eight articles reported sepsis onset prediction 12 or more hours earlier. Five articles reported 24 h earlier, while one article reported 40 h earlier. The majority of the articles reported sepsis onset 2–6 h earlier.

### 4.7. Quality Assessment of Included Studies

Table 2 displays the outcomes of the quality analysis. The quality evaluation was meant to evaluate how well certain prediction models were implemented and reported. Some studies reported a challenge or did not properly report their ML/DL model. Reyna et al. [54] presented the findings of the 2019 PhysioNet/Computing in Cardiology Challenge, which made such an article difficult to assess. The remaining studies had quality ratings ranging from extremely low (meeting 30% of assessment criteria) to very good (more than 80% of assessment criteria). There was no study that met all 16 criteria. Three requirements were met by every single study; all of them had used a sample size of more than 50, used a sepsis definition that adhered to ours in the study design, and reported performance metric(s). One requirement was fulfilled by 95% of the studies, which was reporting sepsis prevalence, while the reporting of the machine learning model in use had been provided in 93% of studies. Out of all the studies, 69% had reported the techniques adopted for handling missing data, 83% of the studies had reported feature engineering techniques used, and 74% of the studies had reported the limitations of their contents. Only a small percentage of research studies (n = 2 (5%)) had deployed their model for prospective study or real-world validation. Only six studies had made their data-cleaning, analysis, and ML/DL code available. Interestingly, 52% of studies had been carried out on publicly available datasets, while only 17% of studies had validated the machine learning models on external datasets, and 21% of studies had reported explainable AI models. Out of all the studies, 46% had reported hyperparameters, 43% had reported techniques to avoid overfitting, and 50% had discussed the clinical applicability of their proposed approaches. We have categorized the articles according to our newly adopted tool (Table 2) into four categories: low quality (LQ) (0–40%), average quality (AQ) (40–60%), above-average quality (AAQ) (60–80%), and high quality (HQ) (80–100%). According to our evaluation criteria, there are 5 HQ, 19 AAQ, 13 AQ, and 5 LQ articles among the 42 articles investigated in this study.

### 4.8. Impact of Funding Source

In our evaluation process, we meticulously assessed the quality of the articles by considering various factors including the availability of funding information. However, our comprehensive analysis revealed that there was no discernible correlation between the presence of funding information and the overall quality of the articles. This observation underscores the independence of funding disclosure from the overall rigor and validity of the research findings, emphasizing the importance of a comprehensive evaluation encompassing various dimensions of article quality, which is reported in Table 2.

## 5. Discussion

This section sheds light on the current state of research, revealing several important insights. However, upon comparison with the existing literature, we acknowledge the existence of other comprehensive systematic reviews and meta-analyses that delve into intricate technical and clinical aspects of sepsis prediction algorithms, thereby offering clinicians an understanding of the challenges and opportunities within this domain. This section also discusses the limitations of our study and challenges and future directions of research in this domain. 

### 5.1. Key Findings

The systematic review conducted a comprehensive search resulting in 1942 articles, from which 42 studies were ultimately selected based on stringent inclusion criteria. These criteria ensured the relevance of the chosen studies, with non-machine learning or -deep learning studies and those with pediatric populations, divergent topics, and non-peer-reviewed designs excluded. The predominance of retrospective designs (n = 38) among the selected studies highlighted the utilization of historical data for analysis, with geographical diversity observed, including a concentration of investigations in the United States (n = 35). The variety of healthcare settings utilized, including ICU (n = 34), ED (n = 11), and general ward data, underscored the complexity of sepsis detection across different contexts. Notably, various datasets were incorporated, with MIMIC-III, PhysioNet/CinC Challenge, and the Emory Healthcare System being primary sources. The adoption of Sepsis-2 and Sepsis-3 definitions for diagnosis indicated the evolving nature of sepsis classification. The range of sepsis prevalence (0.41% to 63.6%) exposed the inherent dataset imbalance, leading many studies to employ data augmentation techniques. The range of parameter utilization and feature importance analysis highlighted the heterogeneity of approaches within the field, emphasizing the ongoing quest for the optimal model.

The analyzed studies reveal a balanced distribution between classical machine learning models (58%) and various deep learning models (42%), indicating a diverse spectrum of approaches. Validation strategies were categorized into train-validation-test split and N-fold cross-validation, with the latter being prominent, especially 10-fold CV. Common metrics like AUROC, Sensitivity, Specificity, and Accuracy were recurrently used. Large disparities are found among sepsis definitions, making it impossible to compare the AUROC value of every study to find the best machine learning model. AUROC values range between 0.80 and 0.97 in different studies. However, comparing model performance based solely on specific metrics can be limiting due to dataset variations. The analysis demonstrates that 71.4% of studies utilized longitudinal data for early sepsis onset prediction, often forecasting sepsis 2–6 h ahead. Quality assessments ranged from extremely low to very good, showcasing the multifaceted nature of article quality. Notably, no study met all 16 quality criteria, highlighting the complexity of evaluation. Funding information showed no consistent correlation with article quality.

A substantial portion of EHR data resides within unstructured clinical notes, encompassing clinician insights not captured by physiological variables. Several studies have explored leveraging natural language processing (NLP) techniques to extract predictive features from clinical notes, targeting sepsis detection [49,52,64,72]. However, most of these investigations have treated NLP features in isolation without integrating them with physiological data. The study conducted by Goh et al. [49] showcases the synergy of NLP and physiological features when early predicting sepsis. This study highlights that combining NLP and physiological attributes yields superior classification performance compared to utilizing NLP or physiological features in isolation.

To ensure a comprehensive evaluation of the quality of the articles, several key aspects should be considered. Firstly, the sample size in a study should be a reasonable size to enhance the statistical robustness of the findings. Additionally, data accessibility and the availability of code should be prioritized to ensure the transparency and replicability of the study. Secondly, for practicality and broader usability, exploring the potential of mobile or web deployment for the developed models can enhance their real-world applicability. Thirdly, the handling of missing data should be addressed using effective strategies to maintain data integrity. Moreover, evaluating the developed models against the prevalence of sepsis is crucial, and meticulous feature engineering should be emphasized to extract relevant insights. Ensuring the adoption of suitable machine learning models and careful tuning of hyperparameters is essential to prevent the risk of overfitting. Furthermore, the transparent reporting of performance metrics enables a clear assessment of model efficacy. External validation is imperative to establish the generalizability of the models, and their explainability should be a priority. Lastly, the discussion should encompass both the clinical implications of the findings and a candid exploration of the study’s limitations.

The quality assessment conducted in this study, as reported in Table 2, reveals that among the studies included, five were designated as high quality, five as low quality, nineteen as above-average quality, and thirteen as average quality.

### 5.2. Summaries of Recent Systematic Reviews in Relevant Fields

Eight systematic reviews and meta-analyses have been identified in the literature (Supplementary Table S5), each focusing on distinct facets of sepsis prediction, precluding direct comparison with our study. Our investigation encompassed a substantial volume of peer-reviewed journal articles, contrasting with some literature sources that also included conference papers. Some articles lacked quality assessment reporting. Nonetheless, our findings align with the majority of these articles except for meta-analysis papers that explored a narrower selection of articles but conducted in-depth investigations.

Jahandideh et al. (2023) [25] explored the use of ML techniques for predicting patient clinical deterioration in hospitals. A total of 29 primary studies were identified, utilizing various ML models, including supervised, unsupervised, and classical techniques. The models exhibited diverse performance, with area-under-the-curve values ranging from 0.55 to 0.99, highlighting the potential for automated patient deterioration identification, although further real-world investigations are needed. Deng et al. (2022) [26] introduced new evaluation criteria and reporting standards for assessing 21 machine learning models based on PRISMA, revealing inconsistent sepsis definitions, varied data sources, preprocessing methods, and models, with AUROC improvement being linked to machine learning’s role in feature engineering. Deep neural networks coupled with Sepsis-3 criteria show promise for time-series data from sepsis patients, aiding clinical model enhancements. Yan et al. (2022) [28] assessed the impact of using unstructured clinical text in machine learning for sepsis prediction. Various databases were searched for articles using clinical text for ML or natural language processing (NLP) to predict sepsis. Findings indicated that combining text with structured data improved sepsis prediction accuracy compared to structured data alone, with varying methods and definitions influencing outcomes. However, the lack of comparable measurements prevented meta-analysis. Giacobbe et al. (2021) [27] focused on the impact of sepsis definition, input features, model performance, and AI’s role in healthcare, with potential benefits in medical decision-making. Sepsis prediction studies in the ICU often rely on MIMIC-II or MIMIC-III data, but insufficient code-sharing hampers reproducibility [18]. Bias in datasets is observed with predominantly Western cohorts, impacting sepsis label creation due to demographic and policy differences. Inconsistent study parameters and metrics prevent meta-analyses, highlighting the need for improved methods and shared code to enhance predictive accuracy and research comparability. Fleuren et al. (2020) [29] demonstrated ML model accuracy in predicting sepsis onset in retrospective cohorts with clinically relevant variables. While individual models outperform traditional tools, study heterogeneity limits pooled performance assessment. Clinical implementation across diverse patient populations is crucial to assess real-world impact. Islam et al. (2019) [23] carried out a meta-analysis investigating ML model performance in predicting sepsis 3–4 h before onset. The ML model in the study outperformed traditional sepsis scoring tools like SIRS, MEWS, SOFA, and qSOFA in recognizing sepsis and non-sepsis cases, with higher ability for sepsis detection. Different datasets showed consistent performance, suggesting machine learning’s potential to reduce sepsis-related mortality and hospital stay by accurately identifying at-risk patients, despite the challenge of sepsis diagnosis due to organ dysfunction and preexisting conditions. Schinkel et al. (2019) [22] identified fifteen articles on sepsis diagnosis with AI models (best AUROC 0.97), seven on mortality prognosis (AUROC up to 0.895), and three on treatment assistance. However, 22 articles exhibited high risk of bias due to the overestimation of performance caused by predictor variables coinciding with sepsis definitions. The authors also reported that AI holds potential for early antibiotic administration, but bias, overfitting, and lack of standardization hinder clinical implementation.

### 5.3. Limitations

This systematic review is subject to publication bias as we have selected studies with significant or positive results. There is a lack of studies reporting negative or null results; therefore, the systematic review’s findings may overstate the effectiveness of machine learning-based early prediction models for sepsis. The studies included in this systematic review vary in terms of patient populations, healthcare settings, study designs, machine learning algorithms used, and outcome measures. This heterogeneity limits the comparability and generalizability of the results, making it challenging to draw definitive conclusions on the performance of different machine learning models used on diverse datasets. Additionally, issues related to data quality, feature availability, and the interpretability of models were identified as challenges in the field. To address these limitations, future studies should focus on the prospective validation of machine learning models, external validation across diverse healthcare systems, and comparative analyses of different algorithms. Efforts to improve data quality, feature engineering, and the interpretability of models are crucial. Furthermore, studies should evaluate the clinical impact of implementing machine learning-based sepsis prediction models, considering patient outcomes, healthcare resource utilization, and cost-effectiveness.

Handling missing data in EHRs, along with noting whether a study is retrospective or prospective, is pivotal. Imputing substantial missing values, even with robust statistical methods, as seen in MIMIC-based studies with up to 50% imputed data, can pose limitations and uncertainties for sepsis prediction—a critical concern for clinicians within machine learning approaches. Assessing the quality and risk of bias in included studies is an essential aspect of a systematic review. However, in this study, we have introduced a 16-parameter quality assessment tool based on previous studies [18,37] to assess the quality of the included articles. Although there are subjective metrics in the scoring system, due to the addition of a large number of metrics, this assessment tool made this evaluation more reproducible compared to earlier systematic reviews.

This systematic review relies on published studies only, which has led to the exclusion of relevant studies available only as preprints. This exclusion could impact the comprehensiveness of the review and potentially overlook recent advancements or findings in the field. Machine learning-based early prediction of sepsis is a rapidly evolving field, and this systematic review has used a cut-off date (March 2023) for the inclusion of studies. Consequently, the review may not capture the most recent advancements or developments in the field, limiting the review’s currency. This systematic review has language restrictions, such as including only studies published in English, which can introduce language bias. Additionally, systematic reviews may only include studies published in indexed journals, leading to potential publication bias by excluding relevant studies from non-indexed sources and conferences.

### 5.4. Challenges and Future Directions

Considering our findings in the context of the broader literature, we recognize several challenges that demand further exploration and investigation. The application of machine learning algorithms in the early prediction of sepsis using EHRs holds great potential for improving patient outcomes [32,70]. By leveraging large amounts of patient data, these models can identify subtle patterns and early indicators of sepsis that may go unnoticed by human clinicians. The early prediction of sepsis enables timely interventions, such as appropriate antibiotic therapy and fluid resuscitation, which can significantly reduce morbidity and mortality rates. However, several challenges need to be addressed for the successful implementation of machine learning-based sepsis prediction models in clinical practice. Here are some key challenges and potential future directions in the field [32,78,79,80]:

- EHR data can be heterogeneous, incomplete, and prone to errors, which poses challenges for accurate prediction models. Future research should focus on improving data quality and standardization, integrating data from multiple sources, and developing techniques to handle missing data effectively.
- EHR data contain a vast number of variables, and not all of them may be relevant for sepsis prediction. Feature selection techniques and advanced representation learning methods, such as deep learning, can help identify the most informative features and extract meaningful representations from the EHR data.
- Sepsis is a relatively rare event compared to non-sepsis cases, leading to imbalanced datasets. Class imbalance can affect model performance, and handling this issue requires techniques such as oversampling, under-sampling, or employing advanced algorithms designed for imbalanced data.
- Machine learning models trained on one healthcare system may not be generalized well to other institutions or patient populations. Future research should focus on the external validation and generalizability of sepsis prediction models across diverse healthcare settings to ensure their real-world effectiveness.
- Black-box machine learning models may lack interpretability, which can limit their adoption in clinical practice. Developing interpretable models and providing explanations for model predictions can enhance trust and facilitate clinicians’ understanding of the underlying reasons for sepsis predictions.
- Early detection and timely intervention are crucial for sepsis management. Future research should focus on developing real-time prediction models that integrate seamlessly into clinical workflows, triggering alerts to clinicians and facilitating prompt action.
- Demonstrating the clinical impact of machine learning-based sepsis prediction models is essential. Prospective validation studies in clinical settings are needed to assess these models’ effectiveness, impact on patient outcomes, and cost-effectiveness compared to existing clinical practices.
- EHR datasets defining sepsis onset time becomes crucial for predictive models’ clinical relevance. The challenge lies in aligning model predictions with actual clinical timelines, considering symptoms’ varying occurrence times. Symptoms manifesting hours before hospital arrival or in different healthcare settings pose complexities in early prediction models’ optimization, which necessitates detailed exploration and discussion regarding patient record alignment and optimization.

## 6. Conclusions

Sepsis exerts significant mortality, morbidity, and healthcare strains, necessitating strategies such as heightened sepsis awareness, early diagnosis, standardized care protocols, and post-sepsis monitoring to alleviate its impact. This systematic review aims to comprehensively synthesize the existing evidence, encompassing diverse classical machine learning (ML) and deep learning (DL) prediction models, performance metrics, key features, and limitations. Notably, around half of the evaluated articles demonstrate above-average quality. The systematic evaluation underscores the potential of ML models (AUROC: 0.80 to 0.97) in predicting sepsis onset using electronic health records (EHRs), often forecasting sepsis emergence 2–6 h beforehand. This research emphasizes the ability of ML-based early sepsis prediction to enhance patient care despite existing challenges. The progressive exploration of this domain promises the development of robust models for clinical integration, ultimately facilitating timely interventions and improved patient outcomes in sepsis management.

## Figures and Tables

**Table 1:** Summary of the selected articles.

Number	Authors	Year of Publication	Dataset(s)	Sepsis Definition	Sample Size	Sepsis Positive Patients	Sepsis Prevalence (%)	Data Types	Number of Variables	Model(s)	AUROC	Retrospective (R) or Prospective (P) ?	Output Metrics	Hours before Onset
1	Gholamzadeh et al. [38]	2023	MIMIC-III	Sepsis-3	1,552,210	27,916	1.79	Demographics, labs, vitals	38	DT, RF, XGB	0.918	R	AUROC, A, P, R, S, F1	-
2	Duan et al. [39]	2023	Shanghai Hospital	Sepsis-2	282	114	40.43	Demographics, labs, vitals	29	Double fusion DL framework	0.92	R	AUC, A, S, R	24
3	Strickler et al. [40]	2023	2019 PhysioNet/CinC Challenge	Sepsis-3	40,336	2932	7.33	Demographics, labs, vitals	40	CSE, MSC-LSTM, MSC-CSE	-	R	A, P, S, R, F1, MCC, Utility	-
4	Zhou et al. [41]	2021	2019 PhysioNet/CinC Challenge	Sepsis-3	40,336	2932	7.33	Demographics, labs, vitals	40	RG, XGB, LR, RF, SVC	-	R	Average regret	-
5	Al-Mualemi and Lu [42]	2021	2019 PhysioNet/CinC Challenge	Sepsis-3	40,336	2932	7.33	Demographics, labs, vitals	40	SVM, RNN-LSTM, Adaptive CNN	-	R	A, S, R	-
6	Rosnati and Fortuin [43]	2021	MIMIC-III	Sepsis-3	22,007	7936	36.06	Labs, vitals	24	MGP-AttTCN	0.660	R	AUC	5
7	Zhang et al. [44]	2021	2019 DII National Data Science Challenge	Sepsis-2	178,843	52,802	29.5	Demographics, labs, vitals	145	GRU, RETAIN, Dipole, LSTM	0.892	R	AUC	4
8	Shashikumar et al. [45]	2021	Emory Healthcare System, UCSD, MIMIC-III	Sepsis-3	85,046	4794	5.64	Demographics, labs, vitals	65	DeepAISE	0.90	R	AUC	4
9	Aşuroğlu and Oğul [46]	2021	MIMIC-III	Sepsis-3	5154	1404	27.24	Vitals	7	DSPA	0.97	R	AUC	6
10	Oei et al. [47]	2021	MIMIC-III	Sepsis-3	48,632	13,935	28.65	Labs, vitals	8	Deep learning	0.86	R	AUC	3
11	Rafiei et al. [48]	2021	2019 PhysioNet/CinC Challenge	Sepsis-3	40,336	2932	7.33	Demographics, labs, vitals	40	Smart Sepsis Predictor	0.86	R	AUC, S, A	12
12	Goh et al. [49]	2021	Singapore government-based hospital	Sepsis-3	5317	327	6.15	Demographic, vitals, notes, labs, treatment	15	SERA algorithm	0.94	P	AUC, R, S	12
13	Bedoya et al. [50]	2020	Duke University Health System	Sepsis-2	42,979	8160	19.0	Demographics, vital, lab, comorbidities, medications	86	RNN, LR, RF	0.88	R	AUROC	5
14	Yang et al. [51]	2020	2019 PhysioNet/CinC Challenge	Sepsis-3	40,336	2932	7.33	Demographics, labs, vitals	168	XGB, EASP	0.85	R	Utility score, AUROC, R, S	6
15	Yuan et al. [52]	2020	Taipei Medical University Hospital	Sepsis-2	1588	444	27.96	Vitals, lab, exam reports, text and images	18	GB	0.89	P	F1 score, A, R, S, PPV	-
16	Kok et al. [53]	2020	2019 PhysioNet/CinC Challenge	Sepsis-3	40,000	2932	7.33	Demographics, labs, vitals	40	TCN	0.99	R	A, R, S, AUROC, AUPRC	-
17	Reyna et al. [54]	2020	Emory Healthcare System, MIMIC-III	Sepsis-3	60,000	2932	7.3	Demographics, labs, vitals	40	ML Models	0.815	R	AUROC, Utility	6
18	Lauritsen et al. [19]	2020	CROSSTRACKS	Sepsis-2	3126	-	-	Diagnoses, labs, vitals, imaging, medications, treatment	6	CNN-LSTM	0.88	R	AUROC, mAP	3
19	Choi et al. [55]	2020	Yonsei University (YU) Severance Hospital	Sepsis (ICD-10)	7743	1136	14.67	laboratory data	36	LR	0.86	R	A, AUROC, R, S, PPV, NPV	-
20	Kim et al. [56]	2020	Tertiary academic hospital	Sepsis-3	49,560	4817	9.7	Demographics, labs, vitals	29	SVM, GB, MARS, LASSO, Ridge, RF	0.93	R	AUROC, R, S, PPV, NPV	-
21	Ibrahim et al. [57]	2020	MIMIC-III	Sepsis-2	13,728	4256	31.0	Vitals, labs	63	RF, GB, SVM	0.96	R	R, S, AUC, PLR, NLR	-
22	Fagerstrom et al. [58]	2019	MIMIC-III	Sepsis-2	59,000	-	-	Demographics, labs, vitals, treatment, medications, diagnoses	-	LSTM	0.83	R	AUROC	40
23	Kaji et al. [59]	2019	MIMIC-III	Sepsis-2	36,176	23,008	63.6	Demographics, labs, vitals, medications	119	LSTM	0.88	R	AUROC, R, PPV	24
24	Giannini et al. [60]	2019	University of Pennsylvania Health System	Sepsis (ICD-9)	172,700	950	0.55	Demographics, labs, vitals	20	RF	0.88	R	AUROC, R, S	6
25	Ginestra et al. [61]	2019	University of Pennsylvania Health System	Sepsis (ICD-9)	162,212	943	0.58	Vitals, comorbidity, labs	-	Early Warning Systems 2.0 (EWS 2.0)	-	P	Response rate	6
26	Schamoni et al. [62]	2019	University Medical Center, Mannheim	Sepsis-3	620	200	32.3	Demographics, Labs, clinical data	55	Non-Linear ordinal regression	0.84	R	AUROC	4
27	Barton et al. [63]	2019	MIMIC-III, UCSF	Sepsis-3	112,952	3673	3.3	Vitals	6	XGBoost	0.88	R	AUROC, R, S	24
28	Delahanty et al. [64]	2019	Tenet Healthcare System	Sepsis-3	2,759,529	54,661	1.98	Demographics, labs, vitals, medications, nursing notes	13	GB	0.97	R	AUROC, R, S, P	24
29	Scherpf et al. [65]	2019	MIMIC-III	Sepsis-2	46,520	2724	7.7	Lab, vitals	10	RNN-GRU, Insight	0.81	R	AUROC	3
30	Bloch et al. [66]	2019	RMC	Sepsis-2	600	300	50	Vitals	4	NN, SVM, LR	0.88	R	AUROC, R, S, PPV, NPV	4
31	VanWyk et al. [67]	2019	MLH System	Sepsis (ICD10)	586	-	-	Lab, vitals	7	RF, RNN	-	R	F2 score, A, R, S, PPV	4
32	van Wyk et al. [68]	2019	MLH System	Sepsis-2	1161	377	32.47	Demographics, vitals	8	RF	-	R	R, F1	5
33	Yee et al. [69]	2019	MIMIC-III	Sepsis-3	9165	872	9.5	Demographics, labs, vitals, diagnosis		Bayesian network	0.81	R	AUROC, R, S, PPV, NPV	24
34	Mao et al. [70]	2018	MIMIC-III, UCSF	Sepsis-2	90,353	1965	9.1	Vitals	30	Insight	0.92	R	AUROC, R, S	4
35	Nemati et al. [32]	2018	Emory Healthcare System	Sepsis-3	27,527	2375	8.6	Demographics, labs, vitals, clinical data	65	AISE	0.85	R	A, R, S, AUC	4
36	Taneja et al. [71]	2017	Carle Foundation Hospital	Sepsis-3	444	76	17.11	Demographics, labs, vitals	15	SVM	0.81	R	R, S, AUC	-
37	Horng et al. [72]	2017	Beth Israel Deaconess Medical Center	Sepsis (ICD-9)	230,936	32,331	14	Vitals, demographics, and notes	12	SVM	0.86	R	AUROC, R, S, PPV	-
38	Kam and Kim [73]	2017	MIMIC-II	Sepsis-2	6362	360	6.2	Demographics, labs, vitals	9	Sepsis LSTM (SepLSTM)	0.92	R	A, R, S, AUC	3
39	Shashikumar et al. [74]	2017	Emory Healthcare System	Sepsis-3	250	100	40.0	Demographics, comorbidity, clinical context, vitals	2	SVM	0.80	P	AUROC, S	2
40	Calvert et al. [75]	2016	MIMIC-II	Sepsis-2	1394	159	11.4	Demographics, labs, vitals	9	InSight	0.92	R	A, AUROC, R, S	3
41	Desautels et al. [76]	2016	MIMIC-III	Sepsis-3	22,853	1840	9.7	Demographics, labs, vitals	8	InSight	0.88	R	AUROC	4
42	Brown et al. [77]	2016	LDS Hospital and Intermountain Medical Center	Sepsis (ICD-9)	132,748	549	0.41	Demographics, labs, vitals	9	NB	0.953	R	AUROC, R, S, PPV, NPV	-

Note: MIMIC—Medical Information Mart for Intensive Care, DT—Decision tree, RF—Random Forest, XGB—Extreme Gradient Boosting (XGboost), ROC—Receiver Operating Characteristic Curve, AUROC—Area under the ROC Curve, A—Accuracy, P—Precession, R—Recall (Sensitivity), S—Specificity, F1—Harmonic mean of precision and recall, AUC—Area under the ROC Curve, CSE—Computational sepsis expert, MSC—Multi-set classifier, MSC-CSE—Multi-set classifier–Computational sepsis expert, MCC—Matthews’s correlation coefficient, RG—Random Guess, LR—Logistic Regression, SVC—Support Vector Classifier, SVM—Support Vector Machine, LSTM—Long short-term memory networks, CNN—Convolutional neural network, MGP-AttTCN—Multitask Gaussian Process and attention-based deep learning model, GRU—Gated Recurrent Unit, RETAIN—REverse Time AttentIoN model (RETAIN), UCSF—University of California, San Francisco, UCSD—University of California, San Diego, MLH—Methodist Le Bonheur Healthcare, DeepAISE—Deep Artificial Intelligence Sepsis Expert, DSPA—Deep SOFA-Sepsis Prediction Algorithm, SERA—Sequential Element Rejection and Admission, EASP—explainable AI sepsis predictor, GB—Gradient Boosting, MARS—Multivariate Adaptive Regression Splines, LASSO—Least Absolute Shrinkage and Selection Operator, PPV—Positive predictive value, TCN—Temporal Convolutional Network, AUPRC—Area under Precision Recall Curve, ML—Machine Learning, mAP—Mean average precision, NPV—Negative predictive value, PLR—Positive likelihood ratio, NLR—Negative likelihood ratio, RNN—Recurrent Neural Network, NN—Neural Network, AISE—Artificial Intelligence Sepsis Expert, NB—Naïve Bayes Classifier, Latter-day Saints (LDS) Hospital and Intermountain Medical Center.

**Table 2:** A quality assessment tool for the included studies.

Number	Authors	Year of Publication	Sample Size > 50	Data Availability	Code Availability	Mobile or Web Deployment	Handling of Missing Data	Sepsis Prevalence	Feature Engineering	Machine Learning Models	Hyperparameters	Sepsis Definition Adhered in Study Design	Valid Methods to Prevent Overfitting	Reporting of Performance Matrix	External Data Validation	Explainability	Limitations of the Study	Clinical Applicability Discussion	Score (%)	Quality Category
1	Gholamzadeh et al. [38]	2023	√	√	×	×	√	√	√	√	×	√	√	√	×	×	√	√	68.75	AAQ
2	Duan et al. [39]	2023	√	×	×	×	√	×	√	√	√	√	√	√	×	×	√	×	56.25	AQ
3	Strickler et al. [40]	2023	√	√	×	×	√	√	√	√	√	√	√	√	×	√	√	×	75.00	AAQ
4	Zhou et al. [41]	2021	√	√	√	√	×	√	√	√	√	√	×	√	×	√	√	√	81.25	HQ
5	Al-Mualemi and Lu [42]	2021	√	√	×	×	√	√	√	√	×	√	×	√	×	×	√	√	62.50	AAQ
6	Rosnati and Fortuin [43]	2021	√	√	√	×	√	√	√	√	√	√	×	√	×	√	×	×	68.75	AAQ
7	Zhang et al. [44]	2021	√	×	√	×	√	√	√	√	√	√	√	√	×	√	×	√	75.00	AAQ
8	Shashikumar et al. [45]	2021	√	√	×	√	√	√	√	√	√	√	√	√	√	√	×	√	87.50	HQ
9	Aşuroğlu and Oğul [46]	2021	√	√	×	×	√	√	√	√	√	√	×	√	×	×	√	√	68.75	AAQ
10	Oei et al. [47]	2021	√	√	×	×	√	√	√	√	√	√	√	√	×	×	×	×	62.50	AAQ
11	Rafiei et al. [48]	2021	√	√	×	×	√	√	√	√	×	√	√	√	×	×	√	√	68.75	AAQ
12	Goh et al. [49]	2021	√	×	×	×	×	√	√	√	×	√	×	√	√	×	√	√	56.25	AQ
13	Bedoya et al. [50]	2020	√	×	×	×	√	√	√	√	√	√	×	√	√	×	√	×	62.50	AAQ
14	Yang et al. [51]	2020	√	√	√	×	√	√	√	√	×	√	×	√	×	√	√	×	68.75	AAQ
15	Yuan et al. [52]	2020	√	×	×	×	√	√	√	√	×	√	×	√	×	×	√	×	50.00	AQ
16	Kok et al. [53]	2020	√	√	×	×	√	√	×	√	√	√	√	√	×	×	√	√	68.75	AAQ
17	* Reyna et al. [54]	2020	√	√	√	×	√	√	×	×	×	√	×	√	×	×	×	×	43.75	AQ
18	Lauritsen et al. [19]	2020	√	×	×	×	√	√	√	√	√	√	√	√	×	×	√	√	68.75	AAQ
19	Choi et al. [55]	2020	√	×	×	×	√	√	√	√	×	√	×	√	×	×	√	×	50.00	AQ
20	Kim et al. [56]	2020	√	×	×	×	√	√	√	√	√	√	√	√	×	×	√	×	62.50	AAQ
21	Ibrahim et al. [57]	2020	√	√	×	×	√	√	√	√	√	√	√	√	×	×	×	×	62.50	AAQ
22	Fagerstrom et al. [58]	2019	√	√	×	×	√	√	√	√	√	√	√	√	√	√	√	×	81.25	HQ
23	Kaji et al. [59]	2019	√	√	√	×	√	√	√	√	√	√	×	√	×	√	√	√	81.25	HQ
24	Giannini et al. [60]	2019	√	×	×	×	×	√	×	×	×	√	×	√	×	×	×	√	31.25	LQ
25	Ginestra et al. [61]	2019	√	×	×	×	×	√	×	×	×	√	×	√	×	×	√	√	37.50	LQ
26	Schamoni et al. [62]	2019	√	×	×	×	√	√	√	√	√	√	×	√	√	×	×	√	62.50	AAQ
27	Barton et al. [63]	2019	√	×	×	×	√	√	√	√	√	√	√	√	√	×	√	√	75.00	AAQ
28	Delahanty et al. [64]	2019	√	×	×	×	×	√	√	√	×	√	×	√	×	×	√	√	50.00	AQ
29	Scherpf et al. [65]	2019	√	√	×	×	√	√	√	√	×	√	×	√	×	×	√	√	62.50	AAQ
30	Bloch et al. [66]	2019	√	×	×	×	×	√	√	√	×	√	×	√	×	×	√	×	43.75	AQ
31	VanWyk et al. [67]	2019	√	×	×	×	×	√	×	√	√	√	×	√	×	×	√	√	50.00	AQ
32	van Wyk et al. [68]	2019	√	×	×	×	×	√	√	√	√	√	√	√	×	×	√	×	56.25	AQ
33	Yee et al. [69]	2019	√	√	×	×	×	√	×	√	×	√	×	√	×	×	×	√	43.75	AQ
34	Mao et al. [70]	2018	√	√	×	×	√	√	√	√	×	√	√	√	×	×	√	√	68.75	AAQ
35	Nemati et al. [32]	2018	√	√	×	×	√	√	√	√	×	√	√	√	√	√	√	√	81.25	HQ
36	Taneja et al. [71]	2017	√	×	×	×	×	√	√	√	×	√	×	√	×	×	×	×	37.50	LQ
37	Horng et al. [72]	2017	√	×	×	×	√	√	√	√	×	√	×	√	×	×	√	×	50.00	AQ
38	Kam and Kim [73]	2017	√	√	×	×	√	√	√	√	×	√	√	√	×	×	√	×	62.50	AAQ
39	Shashikumar et al. [74]	2017	√	×	×	×	×	√	√	√	×	√	×	√	×	×	×	×	37.50	LQ
40	Calvert et al. [75]	2016	√	√	×	×	×	√	×	√	×	√	√	√	×	×	√	×	50.00	AQ
41	Desautels et al. [76]	2016	√	√	×	×	√	√	√	√	×	√	×	√	×	×	√	×	56.25	AQ
42	Brown et al. [77]	2016	√	×	×	×	×	×	√	√	×	√	×	√	×	×	√	×	37.50	LQ
% prevalence of each category			100%	52%	14%	5%	69%	95%	83%	93%	46%	100%	43%	100%	17%	21%	74%	50%		

Note: * this study has reported the outcome of a challenge.

## Data Availability

The authors can provide the dataset upon reasonable request.

## Supplementary Materials

The following supporting information can be downloaded at: https://www.mdpi.com/article/10.3390/jcm12175658/s1, Table S1. Different classical ML and DL models commonly used in the early prediction of sepsis. Table S2. Performance metrics used to interpret results. Table S3. A literature retrieval strategy for sepsis prediction. Table S4. PICOS criteria for the systematic review. Table S5. Summary of systematic review and meta-analysis in the literature. Figure S1. Visual representation of sample size and sepsis-positive patient numbers and percentage. Note: Studies # 1 and 28 were removed while plotting this figure as these datasets are outliers, and including these two studies would make the plot non-representative to most of the studies.
