# Supplementary material for: Machine Learning-Based Early Prediction of Sepsis Using Electronic Health Records: A Systematic Review
Source: J Clin Med. 2023 Aug 30;12(17):5658. doi: 10.3390/jcm12175658 (PMC10488449; doi:10.3390/jcm12175658)
Supplement: Supplementary file 1 [file jcm-12-05658-s001.zip › jcm-2530824-supplementary.pdf]

Supplementary Materials

Supplementary Table S1: Different classical ML and DL models commonly used in the early prediction of sepsis

Type	Machine Learning Model	Description
Classical ML Models	Decision Trees (DT)	Decision Trees (DT) are hierarchical models that use a series of binary decisions to classify or predict outcomes based on input features.
	Random Forest (RF)	Random Forest (RF) is an ensemble machine learning algorithm that builds multiple decision trees and combines their predictions to improve accuracy and robustness.
	Support Vector Machine (SVM)	Support Vector Machine (SVM) is a supervised machine learning algorithm that aims to classify data points by finding the optimal hyperplane that maximizes the separation between different classes.
	Logistic Regression (LR)	Logistic Regression (LR) is a statistical method used for binary classification, predicting the probability of an outcome based on input features.
	Gradient Boosting (GB)	Gradient Boosting (GB) is a machine learning ensemble technique that builds a strong predictive model by combining multiple weak learners in a sequential manner.
	Naïve Bayes (NB)	Naïve Bayes (NB) is a probabilistic machine learning algorithm used for classification tasks, based on Bayes' theorem and the assumption of feature independence.
	k-Nearest Neighbor (kNN)	K-Nearest Neighbors (KNN) is a supervised machine learning algorithm that classifies data points based on the majority class of their k closest neighbors in the feature space.
Deep Learning Models	Recurrent Neural Network (RNN)	A Recurrent Neural Network (RNN) is a type of artificial neural network designed to effectively process sequential and time-dependent data by maintaining memory of previous inputs.
	Long Short-Term Memory networks (LSTM)	Long Short-Term Memory networks (LSTM) are a type of recurrent neural network (RNN) architecture designed to effectively capture and process sequential data, particularly well-suited for tasks involving memory and context preservation.
	Convolutional Neural Network (CNN)	A Convolutional Neural Network (CNN) is a deep learning architecture designed for image and spatial data processing, utilizing convolutional layers to automatically learn hierarchical features from input data.
	Gated Recurrent Unit (GRU)	Gated Recurrent Unit (GRU) is a type of recurrent neural network (RNN) architecture used in machine learning for sequence modeling, designed to capture and utilize long-range dependencies in data sequences while mitigating vanishing gradient problems.
	Neural Network (NN)	A neural network (NN) is a computational model inspired by the structure and functioning of the human brain, used for various tasks such as pattern recognition and data analysis.
	Multitask Gaussian Process and Attention-	MGP-AttTCN is a hybrid model combining Multitask Gaussian Process and Attention-based Deep Learning techniques for predictive tasks.

	based Deep Learning Model (MGP-AttTCN)	
	Temporal Convolutional Network (TCN)	A Temporal Convolutional Network (TCN) is a deep learning architecture designed for sequence data processing, utilizing convolutional layers to capture temporal patterns and dependencies.
	CNN-LSTM	CNN-LSTM is a hybrid neural network architecture that combines Convolutional Neural Networks (CNNs) and Long Short-Term Memory (LSTM) networks for sequential data analysis, leveraging both spatial and temporal features.
	CNN-GRU	CNN-GRU stands for Convolutional Neural Network - Gated Recurrent Unit, a hybrid model combining CNN and GRU architectures for sequential data processing with convolutional and recurrent layers.

Supplementary Table S2: Performance metrics used to interpret results

Measure	Description
Area under curve (AUC) or AUROC (Receiver Operating Characteristics Curve)	Area representing the discriminative power of a test between 0.5 (no discrimination) and 1 (perfect discrimination).
Sensitivity (Recall)	The proportion of affected cases with a positive test result (i.e., a true positive) in reference to all affected cases
Specificity	The proportion of non-affected cases with a negative test result (i.e., true negative) in reference to all non-affected cases
Accuracy	Accuracy is a statistical metric that measures the proportion of correct predictions made by a model out of all predictions.
Precision	Precision is a statistical metric that measures the accuracy of positive predictions made by a model.
F1 Score	The F1 score is a single metric that balances precision and recall for binary classification tasks.
Matthews Correlation Coefficient (MCC)	Matthews Correlation Coefficient (MCC) is a statistical measure that assesses the quality of binary classification predictions, considering true positive, true negative, false positive, and false negative rates.
Mean Average Precision (mAP)	Mean Average Precision (mAP) is a performance metric used to assess the precision and accuracy of information retrieval systems, particularly in scenarios involving ranked search results.
Positive Predictive Value (PPV)	Positive Predictive Value (PPV) is the proportion of true positive cases among the total predicted positive cases in a diagnostic test or model.
Negative Predictive Value (NPV)	Negative Predictive Value (NPV) is a statistical measure that indicates the proportion of true negatives among all negative test results.
Positive likelihood ratio (PLR)	The probability of a true positive test result divided by the probability of a false positive result, with 1 as the lowest limit
Negative likelihood ratio (NLR)	The probability of a false negative test result divided by the probability of a true negative test result, with 1 as a lower limit

Search strategy
A comprehensive literature retrieval is conducted in PubMed, Google Scholar, IEEEXplore and Scopus for papers published between Jun 2016 and Feb 2023. Keywords like sepsis/machine learning/prediction are used for the search.

Supplementary Table S3: A literature retrieval strategy for sepsis prediction

Databases	Search strategy
PubMed	((Sepsis [Title/Abstract]) AND (prediction [All Fields]) AND ((machine learning [Title/Abstract]) OR (deep learning [Title/Abstract]))
Google Scholar	Title, abstract, keywords: sepsis, machine learning, predict
IEEEXplore	(sepsis) AND ((machine learning) OR (deep learning) OR (prediction) OR (electronic health record))
Scopus	Sepsis [Article title] AND prediction [Article title, Abstract, Keywords] AND machine learning [Article title, Abstract, Keywords] OR deep learning [Article title, Abstract, Keywords]

All the included papers are perused by two independent reviewers (KRI and JP), including title-abstract and full text. All disagreements between the two authors are resolved by a third author (MSIS). The chosen papers are limited to languages in English.

Supplementary Table S4: PICOS criteria for the systematic review

Parameter	Inclusion Criteria	Exclusion Criteria
Population	• Adults (age ≥ 18)<br>• Patients admitted to ICU, emergency department and genral ward	• Age<18<br>• Non-human subjects or experimental setups not related to human healthcare.<br>• Not focused on early prediction of sepsis
Intervention	Any	No restriction
Comparator	• At least one classical ML or DL model used<br>• Utilizing EHR data as the primary source of information.	• No ML/DL model<br>• Clinical investigation without the application of ML/DL model<br>• Do not utilize EHRs as a data source.
Outcomes	• Early prediction of sepsis	• Mortality prediction
Study designs	• Retrospective or prospective study<br>• Sample size (>50)	• Studies that focus only on using clinical notes.<br>• Do not report on performance metrics for machine learning models

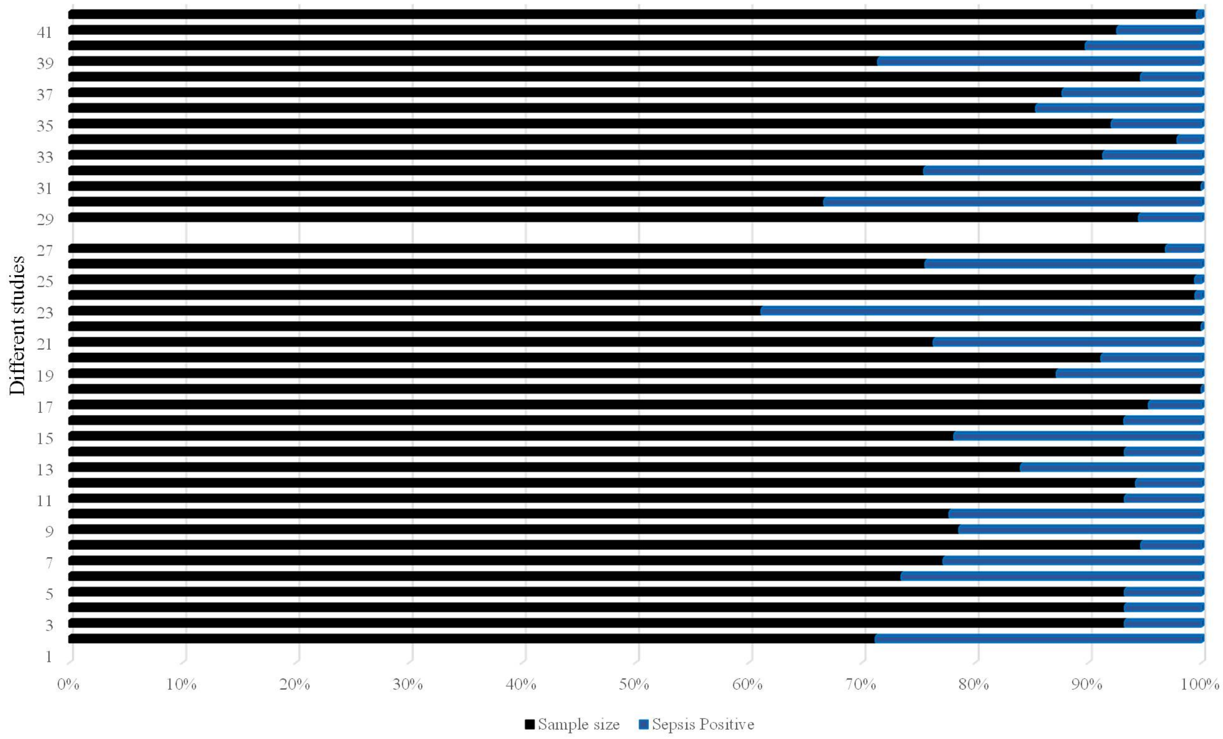

Supplementary Figure S1. Visual representation of sample size and sepsis-positive patient numbers and percentage. Note: Studies # 1 and 28 were removed while plotting this figure as these datasets are outliers, and including these two studies would make the plot non-representative to most of the studies.

Supplementary Table S5: Summary of systematic review and meta-analysis in the literature

SN	References	Journal	Journal Impact Factor	Journal H-index	Citations	Number of articles Reported
1	Jahandideh et al. (2023)	International Journal of Medical Informatics	4.9	122	0	29
2	Deng et al. (2022)	Iscience	5.75	61	14	21
3	Yan et al. (2022)	Journal of the American Medical Informatics Association	7.942	169	15	9
4	Giacobbe et al. (2021)	Frontiers in medicine	3.9	71	27	43
5	Moor et al. (2021)	Frontiers in medicine	3.9	71	76	21
6	Fleuren et al. (2020)	Intensive care medicine	38.9	219	290	24

7	Islam et al. (2019)	Computer methods and programs in biomedicine	6.1	124	149	7
8	Schinkel et al. (2019)	Computers in biology and medicine	7.7	113	75	25

Note: Google scholar is used for citation information
